# The Insecticidal Activity of *Rhinella schneideri* (Werner, 1894) Paratoid Secretion in *Nauphoeta cinerea* Cocroaches

**DOI:** 10.3390/toxins12100630

**Published:** 2020-10-01

**Authors:** Allan Leal, Etiely Karnopp, Yuri Correia Barreto, Raquel Soares Oliveira, Maria Eduarda Rosa, Bruna Trindade Borges, Flávia Luana Goulart, Velci Queiróz de Souza, Manuela Merlin Laikowski, Sidnei Moura, Lúcia Vinadé, João Batista Teixeira da Rocha, Cháriston André Dal Belo

**Affiliations:** 1 Laboratório de Neurobiologia e Toxinologia, LANETOX, Universidade Federal do Pampa, Campus São Gabriel, São Gabriel RS 97307-020, Brazil; allanleal@unipampa.edu.br (A.L.); etielykarnopp@gmail.com (E.K.); barreto78@outlook.com (Y.C.B.); raquelsoaresoliveira@yahoo.com.br (R.S.O.); mariaer2.aluno@unipampa.edu.br (M.E.R.); brunaborges.aluno@unipampa.edu.br (B.T.B.); flaviagoulart.aluno@unipampa.edu.br (F.L.G.); velcisouza@unipampa.edu.br (V.Q.d.S.); luciavinade@unipampa.edu.br (L.V.); 2 Programa de Pós-Graduação em Ciências Biológicas: Bioquímica Toxicológica (PPGBTox), Universidade Federal de Santa Maria (UFSM), Avenida Roraima 1000, Santa Maria RS 97105-900, Brazil; jbtrocha@gmail.com; 3 Laboratório de Biotecnologia de Produtos Naturais e Sintéticos, Instituto de Biotecnologia, Universidade de Caxias do Sul (UCS), Rua Francisco Getúlio Vargas 1130, Caxias do Sul RS 95070-560, Brazil; manusnowwhite@yahoo.com.br (M.M.L.); smsilva11@ucs.br (S.M.)

**Keywords:** toad parotid secretion, natural insecticide, insect behavioral deficits, AChE inhibition, neuromuscular blockade

## Abstract

*Rhinella schneideri* is a common toad found in South America, whose paratoid toxic secretion has never been explored as an insecticide. In order to evaluate its insecticidal potential, *Nauphoeta cinerea* cockroaches were used as an experimental model in biochemical, physiological and behavioral procedures. Lethality assays with *Rhinella schneideri* paratoid secretion (RSPS) determined the LD_50_ value after 24 h (58.07µg/g) and 48 h exposure (44.07 µg/g) (R^2^ = 0.882 and 0.954, respectively). Acetylcholinesterase activity (AChE) after RSPS at its highest dose promoted an enzyme inhibition of 40%, a similar effect observed with neostigmine administration (*p <* 0.001, *n*= 5). Insect locomotion recordings revealed that RSPS decreased the distance traveled by up to 37% with a concomitant 85% increase in immobile episodes (*p <* 0.001, *n* = 36). RSPS added to in vivo cockroach semi-isolated heart preparation promoted an irreversible and dose dependent decrease in heart rate, showing a complete failure after 30 min recording (*p <* 0.001, *n* ≥ 6). In addition, RSPS into nerve-muscle preparations induced a dose-dependent neuromuscular blockade, reaching a total blockage at 70 min at the highest dose applied (*p <* 0.001, *n* ≥ 6). The effect of RSPS on spontaneous sensorial action potentials was characterized by an increase in the number of spikes 61% (*p <* 0.01). Meanwhile, there was 42% decrease in the mean area of those potentials (*p <* 0.05, *n* ≥ 6). The results obtained here highlight the potential insecticidal relevance of RSPS and its potential biotechnological application.

## 1. Introduction

Nowadays, pesticide resistance has become a serious problem, with an estimated 489 species of insects resistant to the most widely used synthetic pesticides, such as pyrethroids, organophosphates and chlorinated hydrocarbons [1,2,3]. With the increased resistance in pest insects in focus, there is an urgency for identifying novel insecticidal molecules with alternative modes of action, preferable of natural origins [2]. 

It has long been recognized that natural product structures present high chemical diversity, biochemical specificity, molecular flexibility and other molecular properties that make them more favorable as lead structures for drug discovery [4]. In addition, all these advantages partially explain why about 60% of the new chemical entities introduced as pharmaceutical choices were directly or indirectly based on natural resources [4].

*Rhinella schneideri*, usually known as cururu toad, is an anuran of the Bufonidae family which has a wide geographic distribution throughout South America, including Brazil, Argentina, Bolivia, Paraguay and Uruguay [5]. Due to its wide global vertical distribution, *R. schneideri* is well adapted to a variety of environmental conditions and can be found in biomes such as Chaco, Cerrado, and Atlantic Forest regions, both in anthropic and natural environments [5]. The successful interaction of Anurans with different environments is a result of very significant morphological adaptations. Perhaps the most important is the presence of granular and serous glands located in the skin, responsible for regulating physiological functions and to protect against predators and harmful microorganisms [6]. One specificity about the Rhinella genus is the presence of well-developed parotid glands located dorsally close to the animal tympanum. The glands are responsible for the production of a very complex and variable dense species-dependent yellowish toxic secretion [7]. Despite its complex biochemical constitution, this toxic secretion is mainly constituted by two chemical groups: the biogenic amines and steroid derivatives (cholesterol, ergosterol, bufotoxins and bufadienolides), that are responsible for digitalis-like action [8,9,10]. Although *Rhinella schneideri* poison constituents still needs further investigation, some researchers carried out previous chemical characterizations of the *R. Shcneideri* paratoid secretion poison. Among the identified compounds, Bufadienolides, such as Bufalin, Marinobufagin and Telocinobufagin, appears to be abundant in *R. schneideri* poison [11].

Recently, few studies have proven the pharmacological potential of the *R. schneideri* toxic secretion [12,13]. However, to the best of our knowledge, no previous work was conceived for investigating the entomotoxic potential of *R. schneideri* toxic secretion. In this work, we identified alkaloids and steroidal compounds present in the paratoid toxic secretion of *R. Schneideri.* Besides, it was shown that the insecticidal activity of *R. Schneideri* paratoid secretion (RSPS) and the association of this effect with a range of neurophysiological and biochemical disturbances affect insect locomotory behavior.

## 2. Results

### 2.1. High Resolution Mass Spectrometry

Analysis of the fractions by high resolution mass spectrometry (HRMS), with electrospray ionization (ESI), in positive and negative modes revealed the presence of the following compounds: *n*-methyl-5-hydroxy-tryptamine, 3,14-dihydroxybufa−20,22-dienolide (Bufalin), 14,15-epoxy-3,5-dihydroxybufa-20,22-dienolide (marinobufagin), 3-(*n*-suberoyl argininyl) marinobufagin, Marinosin (11,19-epoxy-19-methoxy-Telocinobufagin) and 3-(*n*-suberoyl argininyl)-bufalin (Table 1).

### 2.2. Lethality Dose Assay (LD_50_)

**Figure 1:**
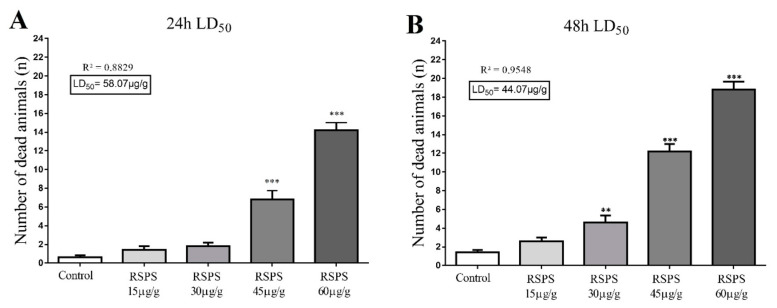
Insecticidal activity of different doses of *R. Schneideri* paratoid secretion (RSPS) in *Nauphoeta cinerea*. (**A**) Dose-dependent increase in mortality after 24 h of exposure by RSPS. Panel (**B**) shows the dose-dependent increase in mortality after 48 h of exposure by RSPS. Statistics were performed by One-way ANOVA followed by Dunnett’s test as post hoc. ** *p* < 0.01, *** *p* < 0.001 (*n* = 5).

The lethality assays performed with RSPS (15, 30, 45 and 60 µg/g) indicated strong insecticidal activity of this poison (Figure 1). Overall, RSPS induced a dose-dependent increase in the number of dead animals. The maximum dose (60 µg/g) led to approximately 58% of lethality for the first 24 h period, and almost 80% after 48 h (*p* < 0.001). The LD_50_ after 24 h was statistically determined at 58.07 µg/g (R² = 0.8829). After 48 h the LD_50_ value was reduced to 44.07 µg/g (R² = 0.9548).

### 2.3. Acetylcholinesterase Activity

**Figure 2:**
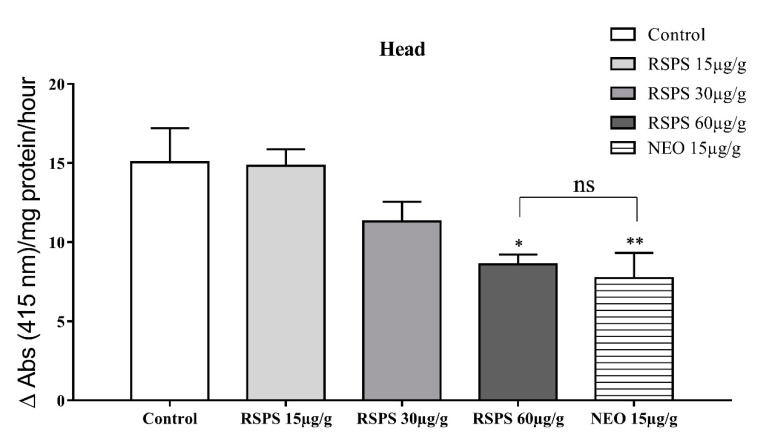
Decrease in acetylcholinesterase activity induced by *R. Schneideri* paratoid secretion (RSPS). Statistical analyses were performed by One-way ANOVA followed by Dunnett’s test. ns: not significant, * *p* < 0.05, ** *p* < 0.01 (*n* = 5).

The analysis of cockroach AChE activity after RSPS administration (15, 30 and 60 µg/g) revealed a dose dependent enzyme inhibition. At the highest dose, there was 40% enzyme inhibition when compared with saline control (Figure 1). The administration of neostigmine 15 µg/g, as a positive control, lead to a similar inhibition in comparison with the most effective dose of RSPS (Figure 2).

### 2.4. Locomotory Activity

**Figure 3:**
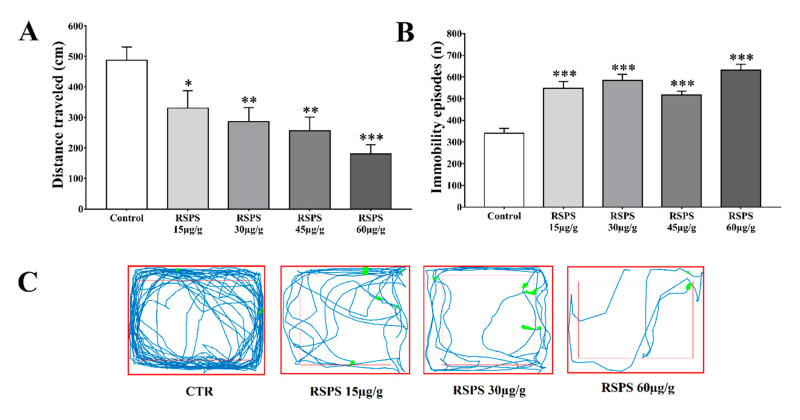
Locomotory deficit induced by *R. Schneideri* paratoid secretion (RSPS) in *Nauphoeta cinerea* cockroaches. Panel (**A**) shows the total distance traveled, (**B**) immobile episodes, and (**C**) the representative traces of the animal tracks. Statistical analysis was performed by One-way ANOVA followed by the Dunnett’s test as post hoc. * *p* < 0.05, ** *p* < 0.01, *** *p* < 0.001 (*n* = 36).

The locomotory recordings performed on cockroaches treated with RSPS (15, 30 and 60 µg/g) revealed a dose-dependent negative relationship. At the highest dose, there was a decrease of about 37% in the total distance traveled (Figure 3A), followed by an 85% increase in immobile episodes (Figure 3B). The exploratory profile of individual cockroaches (Figure 3C) generally revealed a locomotory deficit after RSPS treatment.

### 2.5. Effect of RSPS on Semi-Isolated Cockroach Heart Preparation

**Figure 4:**
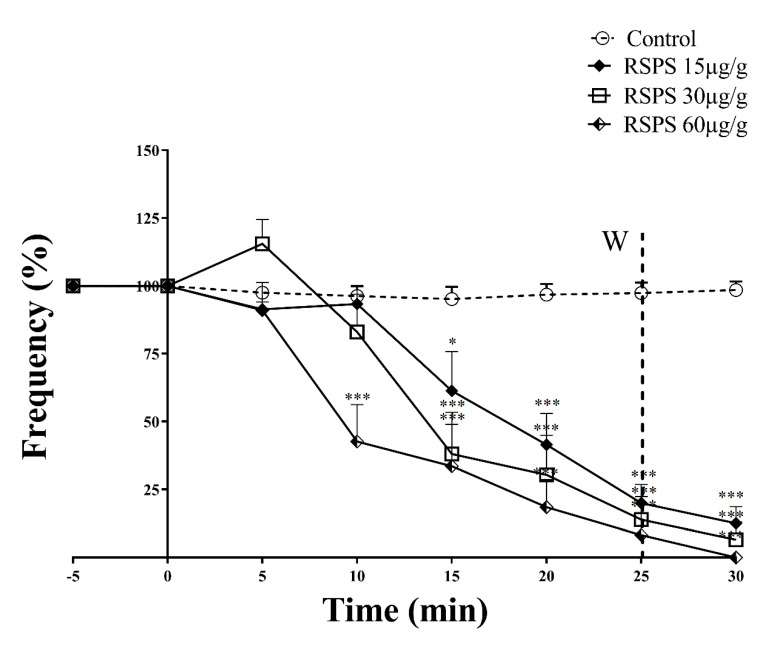
Irreversible negative chronotropic activity induced by *R. Schneideri* paratoid secretion (RSPS) in *Nauphoeta cinerea* semi-isolated heart preparations. W: washout. Statistical analyses were performed by two-way ANOVA followed by the Bonferroni’s test as a post hoc. * *p* < 0.05, *** *p* < 0.001 (*n* ≥ 6).

The addition of RSPS (15, 30 and 60 µg/g) to in vivo cockroach semi-isolated heart preparation revealed an irreversible and dose dependent decrease in heart rates. Even after the wash out there was a complete failure of the heart beats after 30 min recordings, for the highest concentration (Figure 4).

### 2.6. Effect of RSPS at Cockroach Metathoracic Coxal-Adductor Nerve-Muscle Preparation

**Figure 5:**
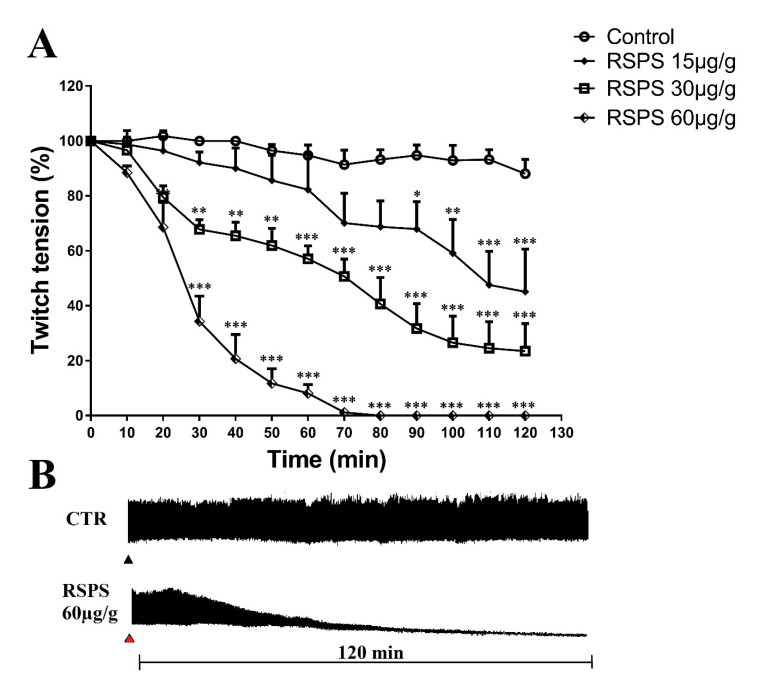
Neuromuscular blockade induced by *R. Schneideri* paratoid secretion (RSPS) at in vivo cockroach neuromuscular preparation. Panel (**A**) shows the graph of percentage of the twitch tension response by time. (**B**) Shows the representative traces of the insect neuromuscular recordings. Statistical analysis was performed by two-way ANOVA followed by Bonferroni’s test as post hoc. * *p <* 0.05, ** *p <* 0.01, *** *p <* 0.001 (*n* ≥ 6). CTR: control saline treatment; ▲: RSPS administration; ▲: Saline administration.

The injection of RSPS (15, 30 and 60 µg/g) induced a dose-dependent neuromuscular blockade. Half inhibition of muscle twitches was observed in approximately 107, 71 and 25 min for each dose, respectively, reaching a total blockage for the highest dose after 70 min of treatment (Figure 5A). The representative traces of the neuromuscular blockade are shown in Figure 5B.

### 2.7. Recordings of Spontaneous Neural Compound Action Potentials (SNCAP)

**Figure 6:**
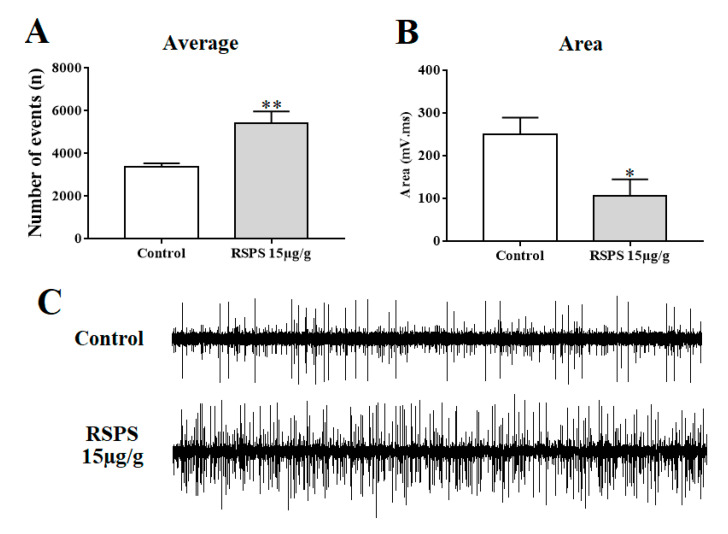
Electrophysiological modulation induced by RSPS in cockroach hair *sensilla* action potentials. Panel (**A**) shows the increase in the frequency of action potentials. Panel (**B**) shows the decrease in the mean area of the action potentials. Panel (**C**) shows representative traces of a control preparation without and after RSPS 15 µg/g treatment. Statistical analysis was performed using Student *t*-test, * *p* < 0.05, ** *p* < 0.01 (*n* ≥ 6).

The activity of RSPS upon the spontaneous activity of the afferent sensorial compound action potentials of cockroach leg *sensilla* was characterized by an increase in the mean firing rates together with a modulation in the potentials kinetics. Thus, the injection of RSPS 15 µg/g induced ~61% increase in the number of spontaneous activity (Figure 6A,C), while there was a 42% decrease in the mean area of those potentials (Figure 6B). Other parameters analyzed, such as duration, time of rise, peak, and decay times were not changed. 

## 3. Discussion

This work brings novel and interesting information about the biological activity of *R. schneideri* parotid secretion (RSPS). In our experimental conditions, RSPS induced insecticidal activity when injected in *N. cinerea* cockroaches. During the progress of the toxic activity, the animals demonstrated sings of neurotoxicity characterized by lethargy and hypokinesis. In this regard, insecticides exert a broad range of effects in insects by several different mechanisms which include: neuroexcitation resulting in hyperactivity, tremor and rigid paralysis due to energy depletion and neuromuscular fatigue, while neuroinhibition results in immobility and paralysis because of possible oxygen deprivation and/or reduced respiratory capacity that ultimately leads to mortality [19]. RSPS also induced a significant inhibition of the cockroach AChE activity, which is a common target for organophosphate pesticides (OPs) [20]. However, OPs and carbamate compounds induce general neuroexcitation before death in insects [21], which was not observed in the onset of RSPS in our experimental conditions. This latter observation indicates that, even though RSPS possess a significant inhibitory activity upon *N. cinerea* AChE, it is not the main toxic target for the poison insecticidal activity. 

Indeed, the recordings of cockroach exploratory/locomotory behavior, after RSPS exposition, showed a decrease in the distance traveled with a concomitant increase in the immobile episodes, suggesting a paralyzing activity of RSPS. In this regard, the insect exploratory behavior is a complex phenomenon, which involves the processing of a series of sensory stimulus. A number of studies indicate that insects use the information acquired from visual, tactile, auditory and olfactory cues to coordinate their movements in an exploratory context [22]. Moreover, aspects of physiological state may affect such decisions, as certain goals may be more or less attractive [23]. Thus, in an intoxicated condition, insects do not tend to prioritize defensive strategies to preserve their lives (e.g., which include to be hidden from a potential predator), as they would do on a normal state [24]. We have previously showed, using *Rhinella icterica* poison, the same deleterious activity upon *N. cinerea* locomotory and exploratory behavior [25]. Interesting from the neurophysiological point of view, with both *R. schneideri* and *R. icterica* poisons, our group showed a detrimental increase in the frequency of leg exteroceptive/proprioceptive sensilla potentials. Thus, we suggest that this excitatory activity of RSPS over stretch neurons of trochanter must be a compensatory stimulatory physiological mechanism, evoked by the depressing activity of the poison upon the central and peripheral neurons [26]. At this point, we cannot rule out about the precise molecular target of RSPS upon sensorial afferents. However, the octopaminergic neurotransmission is a candidate, since it was shown to be involved in the modulation of *N. cinerea* behavior by other toad toxic secretion and OPs [25,27]. In addition, octopamine is a central modulator of insect sensorial responses and positively modulates stretch receptors in *Locusta migratoria* [28].

In our experimental conditions, RSPS also induced a progressive neuromuscular failure in *N. cinerea*. This activity is consonant to the hypokinetic activity of RSPS, and is similar to that produced by *R. icterica* poison using the same experimental model [25]. Recently our group showed, using vertebrate neuromuscular preparations, that RSPS induced an increase in the quantal content of the end plate potentials, suggesting an increase in the amount of ACh release in these terminals [29]. The invertebrate neuromuscular junction differs from vertebrates by using glutamate and GABA as excitatory and inhibitory neurotransmitters, respectively, instead of ACh [30]. From the anatomical point of view, a number of GABAergic interneurons departs from segmental ganglia from ventral nerve cord [31]. The physiological modulation of the insect skeletal muscle also involves the innervations through octopaminergic/tyraminergic (OA/TA) neurons, which receive direct inputs from the dorsal unpaired median neurons (DUM) and the ventral unpaired median neurons (VUM), respectively [32,33,34]. Laboratory assays have shown that the activation of DUM neurons by very low concentrations of octopamine, inhibit the contraction of the locust metathoracic extensor tibia muscle fiber [35]. Thereby, this later information may indicate that the neuromuscular blockade induced by RSPS involves the modulation of the octopaminergic pathways, by the activation of DUM neurons and a consequent GABAergic modulation at neuromuscular junctions. However, it is worth noting that both ACh and GABA also seem to be classical neurotransmitters in mechanosensory neurons [31]. Thus, the increase in firing rates induced by RSPS in leg sensilla could be, at least in part, associated with an anti-AChE activity, but could also be another clue of the increase in GABAergic signaling in peripheral nerves.

The entomotoxic activity of RSPS also involves the insect carditoxicity, as the poison, at any dose assayed, significantly decreased the animals’ heart rates, which is critical in the maintenance of ATP levels [36]. Thus, a negative modulation of heart activity in an intoxicated insect may affect the animal ATP supplies, inducing lethargy by increasing the need of resting mechanisms [36]. Therefore, we suggest that the cardiovascular deficit induced by RSPS, in combination with the other systemic activity induced by the poison—e.g., central and peripheral neurotoxicity—may be the main cause of insecticidal activity. 

Finally, the chemical analyses of RSPS also revealed the presence of bufadienolides (bufalin, marinobufagenin, and marinosin), which are steroidal compounds from toad parotoid secretions known to inhibit the Na^+^/K^+^ ATPase pump [18,37,38,39,40,41]. The inhibitory activity of Na^+^/K^+^ ATPase pump exerts a pro-depolarizing effect in neurophysiological systems [42] that would explain, at least in part, most of the physiological disorders induced by RSPS in N. *cinerea,* including the cardiotoxic activity [25,43].

## 4. Conclusions

This study reveals the insecticidal potential of *R. schneideri* paratoid secretion and showed the presence of alkaloids and steroidal molecules as the main chemical constituents of this poison. The mechanism of entomotoxic activity involves cardiotoxicity, neuromuscular failure, electrophysiological and biochemical alterations, together with the disturbance of the cockroach behavior. A future laboratory attempt focused on the isolation and functional characterization of the chemical compounds present in the poison will help to improve the knowledge about the mechanism for insecticidal activity.

## 5. Materials and Methods 

### 5.1. Experimental Animals

All experiments were performed on adult N. *cinerea* cockroaches from both sexes. Animals were reared with water and food ad libitum at controlled temperature and lighting (± 26 °C and 12 h light/dark cycles). Details about the diet are provided in the Supplementary Materials. To ensure standard experimental conditions, all experiments were performed during the same period of the day (from 9:00 a.m. to 4:00 p.m.). Project identification code: 20181112115231, Federal University of Pampa (UNIPAMPA). Brazil does not have a current ethical regulation in terms of using invertebrates as scientific experimental models.

### 5.2. Rhinella Schneideri

*R. schneideri* toads of both sexes were collected at São Gabriel (30° 20.0268’S; 54° 21.7549´O) in the state of Rio Grande do Sul, Brazil. Animals capture was performed under prior authorization of the Brazilian Biodiversity Information and Authorization System (SISBIO): collector license No. 24,041–2.

### 5.3. Rhinella Schneideri Parotid Secretion (RSPS )

The poison was extracted from the parotid glands by manual compression [25]. The extraction product was characterized by a yellowish color and viscous texture. The fresh poison was then lyophilized (Liobras, Liotop K105, Sao Paulo, Brazil) immediately after the extraction procedure, resulting in a powdered extract.

### 5.4. Biological Assays

For the analysis of *R. schneideri* biological activity, cockroaches were injected under the third abdominal segment, directly into the hemocoel, using a Hamilton syringe^©^ in a 20µl final volume, unless otherwise specified. In all experiments, control groups received only saline solution (0.9%NaCl). Treated animal groups were previously weighed and received saline solution containing RSPS, according to the desired concentration (15, 30, 45 and 60 µg/g of animal).

### 5.5. Reagents and Solutions

All reagents and solutions used were of high purity, purchased from Sigma-Aldrich, Life Technologies, Merck, Roche, or BioRad.

### 5.6. Fractionation and Identification

#### 5.6.1. Chemical fractionation

In order to improve the chemical identification of the RSPS constituents, a preliminary fractionation assay was conducted with Ethyl acetate and hexane. To accomplish this, the dried poison was suspended in distilled water and mixed in 50% (*v*/*v*) organic solvents, individually. The aqueous phase was reserved and called SCH Aq, while the phase recovered from ethyl acetate was named SCH Et. Due to the low resulting recovery, the hexanic extract chemical constituents were possible to identify. 

#### 5.6.2. High Resolution Mass Spectrometry

For mass spectrometry, the whole poison (SCH) and fractions were dissolved in a solution consisting of 50% (v/v) chromatographic grade acetonitrile (Tedia, Fairfild, OH, USA) and 50% (v/v) deionized water, to which 0.1% formic acid and 0.1% ammonium formate was added for analysis in positive and negative electrospray ionization modes (ESI(+) and ESI(-), respectively). 

The individual solutions were infused directly into the ESI source via a syringe pump (Harvard Apparatus, Hamilton, Reno, NV, USA), at a flow rate of 180μL/min. The ESI(+)-and ESI(-) mass spectrometric (MS) and tandem MS-MS profiles were acquired using a hybrid high-resolution and high-accuracy (5μL/L) micrOTOF-Q mass spectrometer (Bruker Scientific^®^, Billerica, MA, USA) under the following conditions: capillary and cone voltages were set to +3500 and +40 V, respectively, with a desolvation temperature of 200 °C. The collision-induced dissociation energy (CID) for the ESI(+) MS-MS was optimized for each component. The diagnostic ions were identified by comparison of their dissociation patterns, exact mass and isotopic ratio, with compounds identified in previous studies. For data acquisition and processing, time-of-flight (TOF) control and data analysis software (Bruker Scientific^®^) were used.

The data were collected in the 70–1200 *m*/*z* range, at a rate of two scans/s, providing 50,000 full width at half maximum (FWHM) resolution at 200 *m*/*z*. No important ions were observed below 90 *m*/*z* and above 950 *m*/*z*, so the ESI(+)-MS data are shown for the range of 90–950 *m*/*z*. 

### 5.7. Lethality Dose Assay (LD_50_)

The insecticidal activity assays were based on those described by Kagabu et al. [44] and validated by Carrazoni et al. [45] in *Nauphoeta cinerea* cockroaches. Four statistical blocks containing 24 animals in each group were used. Dead individuals were counted after 24 and 48 h. The lethal dose that killed 50% of the population (LD_50_) was found by linear regression.

### 5.8. Acetylcholinesterase Activity

The AChE activity was evaluated one hour after the in vivo treatment. Each group, composed of 3 animals (*n* = 5), was frozen at −20 °C to stop any enzyme activity. Subsequently, animals had their heads removed and placed in a 500 µL of potassium phosphate buffer (KH_2_PO_4_ 0.1M and K_2_HPO_4_ 0.1M pH7) together with a metal sphere for the homogenization process (3 times, 2000 rpm for 1 min) using PowerLyzer^®^ homogenizer. The homogenate was centrifuged (10,000 rpm for 5 min at 4 °C). Afterwards, the supernatant was collected, and the protein amount was quantified and the cholinesterase activity was measured according to [46].

### 5.9. Assay for Locomotory Activity

The locomotor parameters were evaluated as described by Leal et al. [25], with a few modifications. Control and treatment groups were injected 20 min before locomotor patterns were evaluated. Animal tracking was performed using the software idTracker (Stoelting, CO, USA). Behavioral patterns were obtained through mathematical analysis using Matlab^®^ software (30 days free-trial license) together with an *ad-hoc* script specially developed for this purpose.

### 5.10. Semi-Isolated Cockroach Heart Preparation

The activity of RSPS upon *Nauphoeta cinerea* cardiac rhythm was evaluated as described by Rodríguez et al. [47], with a few modifications. Animals were previously anesthetized by chilling (5–7 min) and then fixed ventral side up on a dissection plate, were their viscera were carefully moved, allowing for cardiovascular system visualization. RSPS, as well as control saline solution, were added directly to the abdominal cavity. The preparation was bathed with 200 µl saline solution and the cardiac rhythm was recorded during the first 5 min to ensure the preparation stability. Following this period, the control solution was replaced by 200 µL treatment solution containing RSPS at desired concentrations and the recordings accomplished for 25 min. After that, the preparation was washed out with saline solution and monitored for a further 5 min. The “Y” axis describes the percentage (%) of the average heart rate in the control and treated groups. Control group was considered 100%.

### 5.11. Metathoracic Coxal-Adductor Nerve-Muscle Preparation

The in vivo metathoracic coxal-adductor nerve-muscle preparation (MCANM) was used in order to evaluate the activity of RSPS on insect peripheral nervous system. The preparation was assembled as described by Martinelli et al. [48]. Briefly, animals were previously anesthetized by chilling (5–7 min) and then fixed ventral side up in a stage covered with soft rubber. After immobilization, one of the third methatoracic legs was tied up and connected to a 1 g isometric force transducer (AVS Instruments, São Carlos, SP, Brazil). A bipolar electrode was inserted into the abdominal region to touch the fifth nerve (which includes the motor axon of the muscle) in order to provide indirect electrical stimulation (0.5 Hz/5 ms). The muscle twitches were recorded for 120 min, by means of a data acquisition and analysis system (AQCAD and ANCAD, respectively) (AVS Instruments, São Carlos, SP, Brazil).

### 5.12. Recordings of Spontaneous Neural Compound Action Potentials (SNCAP)

The ex situ extracellular recording of leg sensilla spontaneous action potentials was performed as described by Leal et al. [25]. In this set of experiments, cockroaches were previously treated with different doses of RSPS, 20 min before being anesthetized by cooling, and had one of the third methatoracic pairs of legs removed and pinned to a cork board with the aid of a pair of Ag/AgCl electrodes. The action potentials were than recorded using the Neuron SpikerBox system (Backyard Brains, USA). The action potentials were visualized, recorded and retrieved for later analysis using the BYB Spike Recorder software (Backyard Brains, USA). Data analysis was performed by WinWCP software (John Dempster, University of Strathclyde, Scotland).

### 5.13. Statistical Analysis

The results were expressed as mean ± SEM. To perform the comparison between two means, Student t-test was employed. To compare averages of two or more groups, where “Y” is usually one variable, one-way ANOVA was performed followed by Tukey (all groups were compared with each other) or Dunnet (the groups were compared with a positive control saline). To compare more than two averages, and if the “Y” had more than one variable, two-way ANOVA, followed by the Bonferroni as a post hoc was applied (groups were compared with a positive control). All statistical analyses were performed by using GraphPad Prism 7.0. (Software, San Diego, CA, USA). The values were considered significant when *p* ≤ 05. 

## Figures and Tables

**Table 1:** Chemical identification of *Rhinella schneideri* paratoid secretion (RSPS) by high resolution mass spectrometry, in positive and negative electrospray ionization modes (ESI (+) and ESI (-), respectively). SCH: Total Poison, SCH Aq: Aqueous phase, SCH Et: Ethyl acetate phase.

Entry	Precursor Ion *m*/*z*	Extract	Identification	Elem. Comp.	Diff Ppm	Comp. Type	Ref.
Extracts analysis in positive mode ESI (+)							
**1**	191.1173	SCH Aq	*n*-methyl-5-hydroxy-tryptamine	C_11_H_14_N_2_O	5.76	Alkaloid	[14]
**2**	387.2523	SCH Aq	3,14-dihydroxybufa-20,22-dienolide (Bufalin)	C_24_H_34_O_4_	3.09	Steroid	[15]
**3**	401.2321 401.2325 401.2322	SCH Aq SCH SCH Et	14,15-epoxy-3,5-dihydroxybufa-20,22-dienolide (marinobufagin)	C_24_H_32_O_5_	1.74 0.75 1.49	Steroid	[15]
**4**	713.4089	SCH Aq	3-(*n*-suberoyl argininyl) marinobufagin; (Mari- nobufotoxin)	C_36_H_53_N_4_O_9_	5.05	Steroid	[15,16,17]
**Extracts analysis in negative mode ESI (-)**							
**5**	445.2220 445.2269	SCH Aq SCH Et	11,19-epoxy-19-methoxy-Telocinobufagin (Marinosin)	C_25_H_34_O_7_	1.34 9.65	Steroid	[18]

Elem. comp.—elemental composition. Comp. type–component type (broad classification).

## Supplementary Materials

The following are available online at https://www.mdpi.com/2072-6651/12/10/630/s1, A text file containing a brief explanation of the experimental animal maintenance and the nutritional information of the feed is attached.
