# Supplementary material for: The Insecticidal Activity of Rhinella schneideri (Werner, 1894) Paratoid Secretion in Nauphoeta cinerea Cocroaches
Source: Toxins (Basel). 2020 Oct 1;12(10):630. doi: 10.3390/toxins12100630 (PMC7601029; doi:10.3390/toxins12100630)
Supplement: Supplementary file 1 [file toxins-12-00630-s001.pdf]

Supplementary Materials: The Insecticidal Activity of Rhinella schneideri (Werner, 1894) Paratoid Skin Toxic Secretion in Nauphoeta cinerea Cocroaches

Allan Leal, Etiely Karnopp, Yuri Correia Barreto, Raquel Soares Oliveira, Maria Eduarda Rosa, Bruna Trindade Borges, Flávia Luana Goulart, Velci Queiróz de Souza, Manuela Merlin Laikowski, Sidnei Moura, Lúcia Vinadé, João Batista Teixeira da Rocha and Cháriston André Dal Belo

Maintenance

All experimental animals were fed with dog Chow.

Nutritional information:

Crude Protein (min.) 180g/kg (18%)
Etheric Extract (min.) 50g/kg (5%)
Linolenic acid (min.) 2000mg/ kg,
Linoleic acid (min.) 10g/kg (1%)
Calcium (max.) 23g/kg (2.3%)
Calcium (min.) 10 g/kg (1%)
Phosphorus (minimum) 8000mg/kg
Fibrous Matter (max.) 60g/kg (6.0%)
Mineral Matter (max) 100g/kg (10%)
Saponin 7mg/kg
Humidity 120g/kg (12%)
Sodium (min.) 2500mg/kg

Highlights

• RSPS induced a dose-dependent increase in the number of dead animals. LD_{50} values: 24h (58.07μg/g) and 48h (44.07μg/g) of exposure.

• RSPS induced 40% of AChE inhibition.

• RSPS induced 37% decrease in the insect distance traveled with a concomitant 85% increase in immobile episodes.

• RSPS induced an irreversible and dose dependent decrease in heart rate, reaching a complete failure.

• RSPS induced a dose-dependent neuromuscular blockade, reaching a complete blockade.

• RSPS induced 61% increase in the number spontaneous action potentials and 42% decrease in the mean area of those potentials.
